# Total IgE and eotaxin (CCL11) contents in tears of patients suffering from seasonal allergic conjunctivitis

**DOI:** 10.1007/s00417-014-2683-6

**Published:** 2014-06-12

**Authors:** Simone Eperon, Marouen Berguiga, Pierluigi Ballabeni, Catherine Guex-Crosier, Yan Guex-Crosier

**Affiliations:** 1 Ocular Immunology, Jules Gonin Eye Hospital, University of Lausanne, 15, Av. de France, 1000 Lausanne 7, Switzerland; 2 Ophthalmology Department, Percy University Hospital, Clamart, France; 3 Institute of Social and Preventive Medicine, Centre Hopitalier Universitaire Vaudois, University of Lausanne, Lausanne, Switzerland

**Keywords:** Tear, Allergic conjunctivitis, IgE, Eotaxin, Conjunctival papillae

## Abstract

**Background:**

To prospectively investigate patients with seasonal allergic conjunctivitis (SAC) during the pollen season and test associations between tears total IgE, eotaxin concentrations, and SAC severity.

**Methods:**

Enrolled patients presented ocular symptoms and clinical signs of SAC at the time of presentation. Ocular itching, hyperaemia, chemosis, eyelid swelling, and tearing were scored, and the sum of these scores was defined as the clinical score. Conjunctival papillae were separately graded. We measured eotaxin concentration in tears by an enzyme-linked immunosorbent assay (ELISA) and total tear IgE by Lacrytest strip.

**Results:**

Among thirty patients (30 eyes), 11 showed neither tear IgE nor tear eotaxin, while 15 out of 19 patients with positive IgE values presented a positive amount of eotaxin in their tears (Fisher’s test: *p* < 0.001). The mean eotaxin concentration was 641 ± 154 (SEM) pg/ml. In patients with no amount of tear IgE, we observed a lower conjunctival papilla grade than in patients whose tears contained some amount of IgE (trend test: *p* = 0.032). In the 15 patients whose tear eotaxin concentration was null, tear IgE concentration was 5.3 ± 3.5 arbitrary units; in the other 15 patients whose eotaxin was positive, IgE reached 21 ± 4.3 arbitrary U (Mann–Whitney: *p* < 0.001). We measured 127 ± 47 pg/ml eotaxin in patients with no history of SAC but newly diagnosed as suffering from SAC, and 852 ± 218 pg/ml eotaxin in patients with a known SAC (*p* = 0.008). In contrast, tear IgE concentrations of both groups did not differ statistically significantly (*p* = 0.947).

**Conclusions:**

If IgE and eotaxin secreted in tears are major contributors in SAC pathogenesis, they however act at different steps of the process.

## Introduction

Allergic diseases, such as asthma, dermatitis, food allergy, and allergic conjunctivitis, affect one third of the population [1, 2]. These diseases have an important impact on patient quality of life and comfort, and result in a burden on the economy through healthcare costs and productivity reduction. Among ocular allergies, seasonal allergic conjunctivitis (SAC) affects a great number of patients. Understanding better the pathogenesis of SAC could help in treating patients [3, 4].

Tear film plays an important role in maintaining a barrier defence at the ocular surface. During SAC, specific Immunoglobulin E (IgE) molecules are secreted in tears and combine with Fc receptors on mast cells [2, 5, 6]. In cases of re-exposure to allergen, the specific IgE on the surface of mast cells catch the allergen, leading to mast cell degranulation and mediator release, such as histamine, causing chemosis and ocular itching. Concentrations of many cytokines and chemokines increase in tears, among them, eotaxin which plays a major role in eosinophil and lymphocyte recruitment [2, 5, 6].

In ocular allergic reactions, some amounts of IgE were measured in tears of patients suffering from SAC, perennial allergic conjunctivitis (PAC), vernal (VKC) and atopic keratoconjunctivitis (AKC) [7–10].

Eotaxin, also called CC chemokine ligand 11 (CCL11), was found in basal tears of healthy subjects [11, 12] and was constitutively expressed in their conjunctiva [13]. A significant increase in eotaxin level has been demonstrated in patients suffering from either SAC during the pollen season or VKC [14, 15]. Eotaxin concentration in tears of SAC patients was significantly higher in season than out of season, when patients were tested at both seasons [14].

Our goal was to analyse the relationship between total IgE level in tears, eotaxin concentration in tears, the clinical score of SAC and papilla grade in the upper tarsal conjunctiva. To our knowledge, this is the first time that both eotaxin and IgE levels have been concomitantly measured in tears of SAC patients during the in season.

## Materials and methods

### Patients and anamnesis

Sixty patients were recruited by the Ocular Immuno-Infectiology Unit of our hospital in this prospective study from March 2007 till August 2007 and from March 2008 till August 2008, which corresponds to the pollen season.

The study was performed in accordance with ethical standards (Declaration of Helsinki, Human Research Ethics Committee in Lausanne). All participants gave their informed consent. A sheet form with questions made it possible to draw patient medical history and both general and ocular anamnesis. Each patient presented with ocular symptoms of SAC, such as itching, a mandatory symptom, redness, tearing, or ocular pain and clinical signs, such as mucous discharge, chemosis, or palpebral papillae. Some patients were suffering from a known SAC diagnosed at least 1 year ago, i.e., pollen allergy. The others had not been diagnosed yet as suffering from SAC at the time of presentation, but were diagnosed as new SAC cases after their visit to the hospital. Ocular exclusion criteria were PAC, VKC, AKC, parasitic, bacterial or viral conjunctivitis, glaucoma, rosacea, dry eye syndrome, and ocular wound. Systemic exclusion criteria were infections, atopic dermatitis, eczema, nettle rash, and systemic diseases. Patients were asked to abstain from taking anti-inflammatory and anti-histaminic drugs. A wash out period of 10 days was considered for these drugs before the enrolment. None of the subjects wore contact lenses, and none of them used topical drugs or nasal drops. Given all these criteria, it was possible to enrol 41 patients for tear collection.

Each individual underwent complete ophthalmic slit-lamp examination by the same ophthalmologist (MB). Among retained patients, some were suffering from a known SAC; the others presented with no known ocular pathology, but their pathology was finally diagnosed as SAC.

Ocular itching, conjunctival hyperaemia, conjunctival chemosis, eyelid swelling, and tearing were scored according to a semi-quantitative scale from 0 to 3. The clinical score was the sum of the scores for each criterion, where 0 represented no symptom/sign and 15 the maximum. Papillae were separately graded in the upper tarsal conjunctiva by slit-lamp examination according to a semi-quantitative scale from 0 to 4 based on standard photos [16].

### Eotaxin in tears

Around 8 μl of tear samples of each patient was necessary for analysis. To obtain unstimulated basal tears, the tear samples were collected with glass microcapillary tubes in the temporal part of the fornix of the inferior eyelid. No anaesthetic was used. Tear samples were immediately centrifuged at 4 ºC to remove cells, transferred to new tubes and frozen at –80 °C.

Eotaxin concentrations were measured by a home-made enzyme-linked immunosorbent assay (ELISA) [14]. A plate was coated with a capture antibody, the purified mouse anti-human eotaxin monoclonal antibody 3C7 (Becton Dickinson: Pharmingen, # 23051D). A serial dilution of recombinant human eotaxin between 16 and 500 pg/ml and dilutions of samples were prepared in 0.05 % Tween and 1 % Bovine Serum Albumin in PBS. After washing and blocking of non-specific binding, eotaxin dilutions in duplicate and samples were added. The plate was washed and a detecting antibody, the biotinylated mouse anti-human eotaxin monoclonal antibody 10C11 (BD Pharmingen, # 23252D), was added. The plate was washed and streptavidin-alkaline phosphatase conjugate (BD Pharmingen # 554065) was added. Alkaline phosphatase yellow liquid substrate (Sigma, # A3469) was applied, and the OD was read at 405 nm with a reference filter at 490 nm. Eotaxin concentration in the samples was read from the standard curve of eotaxin. The limit of the sensitivity of the test was 60 pg/ml of eotaxin. Samples containing <60 pg/ml eotaxin were considered eotaxin-negative. Samples ≥60 pg/ml were considered eotaxin-positive.

### Total IgE in tears

After tear collection with the microcapillary, the Lacrytest (Adiatec SA, Diagnostic and Biotechnologies, Nantes, France) strip was set in the external third of the inferior lid, away from the cornea, until a red line appeared in the control field. The strip was then soaked in the commercial vial for at least 10 min, and removed. Internal control on the strip made it possible to ensure that the result was valid. Patients presenting invalid results were withdrawn from the study. The result was read in the IgE reactive field where the signal intensity depends on total IgE level. No red line in this field was an indication that the sample was negative, IgE level being <2.5 kUI/ml. A red line indicated a positive result, IgE being ≥2.5 kUI/ml. We obtained semi-quantitative IgE concentrations to which we attributed arbitrary units (U): <2.5 kUI/l (=0 arbitrary U), between 2.5 and 10 kUI/l (=5 arbitrary U), between 10 and 40 kUI/l (=20 arbitrary U) and >40 kUI/l (=50 arbitrary U).

### Statistics

**Fig. 1:**
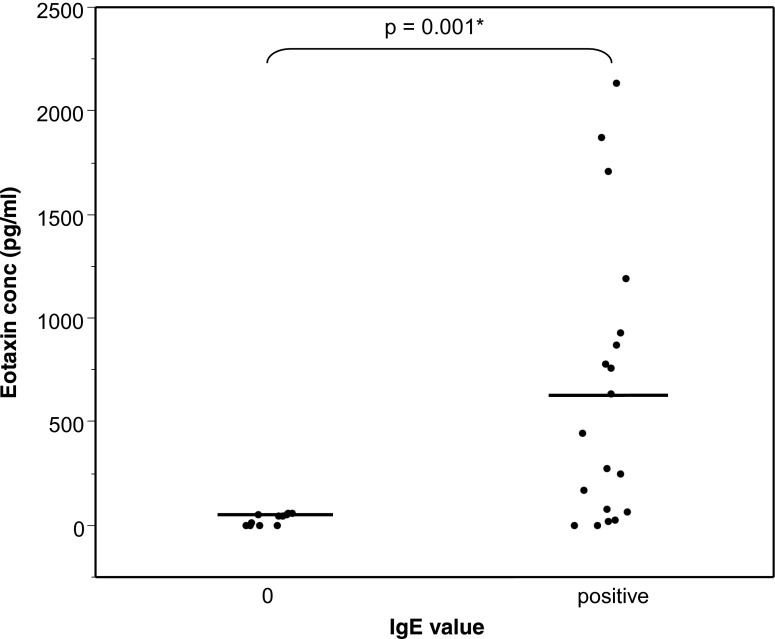
Tear eotaxin concentration and absence (*0*) or presence of IgE in tears. *Bars* = means

**Fig. 2:**
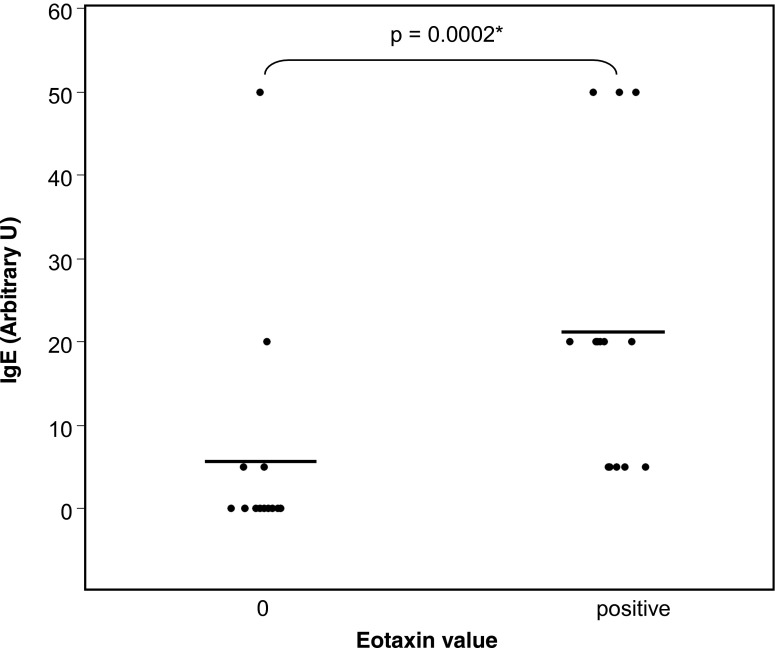
Total tear IgE concentration and absence (*0*) or presence of eotaxin in tears. *Bars* = means

**Fig. 3:**
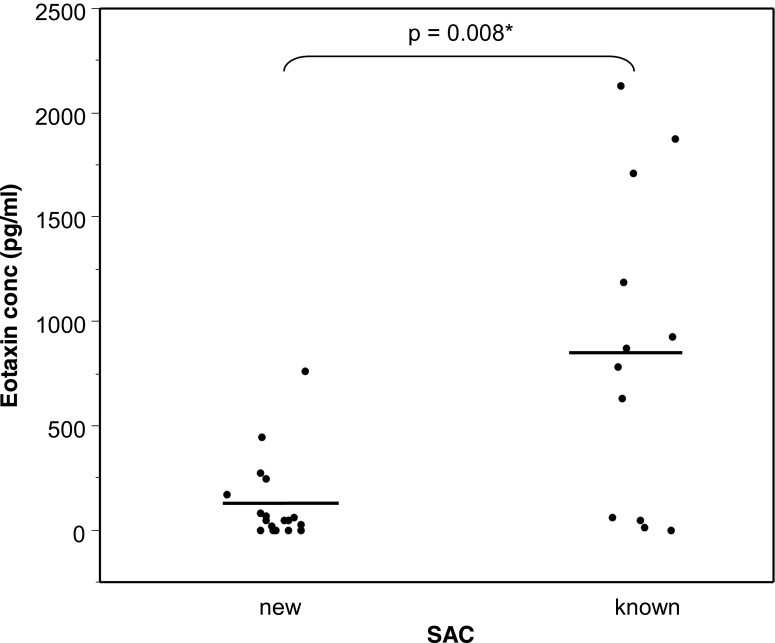
Eotaxin concentration in tears of patients presenting signs and symptoms of SAC. Some patients had a history of SAC (known); for the others, SAC was newly diagnosed following the visit at the hospital (new). *Bars* = means

**Fig. 4:**
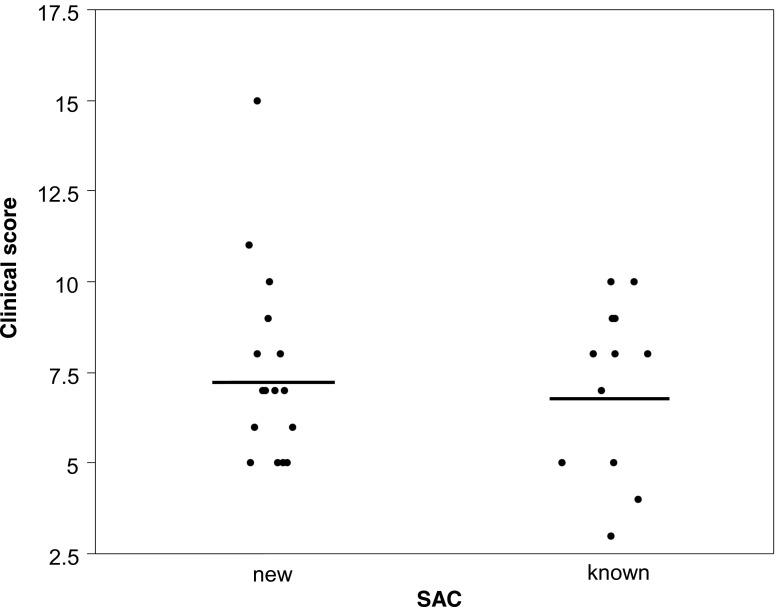
Clinical score of allergic conjunctivitis in patients presenting signs and symptoms of SAC. Some patients had a history of SAC (known); for the others, SAC was newly diagnosed following the visit at the hospital (new). *Bars* = means, *p* = 0.797

To compare the proportion of patient tears, we drew a contingency table and used the Fisher’s exact test. We compared the parameters by means of the Wilcoxon-Mann–Whitney test (Figs. 1, 2, 3 and 4). Values are means ± SEM. Differences were considered to be statistically significant at *p* < 0.05. The statistical package Stata, version 12 (StataCorp LP, College Station, TX, USA) was used.

## Results

Thirty patients (30 eyes, Table 1) could be included in this study for data analysis after exclusion of 11 patients presenting either invalid IgE results or too few tears to allow eotaxin measurement. All patients disclosed ocular symptoms and signs compatible with SAC. None of the patients suffered from PAC, VKC, AKC, parasitic, bacterial, or viral conjunctivitis. Eight patients were men and 22 women. The mean age was 40.8 ± 2.9 (SEM) years (range 18–72 years). For 12 patients, we had a record and history of known SAC, diagnosed at least 1 year ago. Three patients were also suffering from asthma (# 5, 11, and 24).

**Table 1:** Patient characteristics of the prospective study. Patients presented seasonal allergic conjunctivitis (SAC) symptoms. For some patients, SAC had already been diagnosed (+); for the others, SAC was unknown at time of presentation (−)

Patient #	Age	Gender	History of SAC	Clinical score	Papilla grade
1	49	F	–	6	1
2	33	F	+	10	2
3	49	F	–	5	1
4	54	F	+	4	2
5	36	F	+	5	2
6	47	F	+	9	2
7	54	F	+	9	2
8	44	F	+	7	2
9	39	M	–	7	2
10	18	M	+	3	3
11	23	F	+	8	2
12	18	M	–	6	2
13	27	F	–	7	2
14	61	M	–	5	1
15	72	M	–	6	1
16	24	F	+	5	1
17	62	F	–	9	3
18	35	F	–	7	3
19	23	F	–	5	1
20	40	F	–	8	0
21	18	M	–	8	2
22	60	M	–	11	3
23	18	M	–	15	3
24	24	F	–	5	2
25	55	F	+	10	2
26	60	F	–	10	2
27	35	F	–	7	2
28	55	F	+	8	0
29	31	F	–	5	1
30	59	F	+	8	2

The contingency Table 2 presents patients whose tears contained zero arbitrary U or a positive value of IgE (5, 20, or 50 arbitrary U), and no significant amount of eotaxin or a positive value of eotaxin (≥60 pg/ml). One hundred percent of patients with tear IgE = 0 showed tear eotaxin = 0, whereas among patients with a positive value of IgE, 79 % patients showed a positive value of eotaxin (Fisher’s exact test, two-tailed; *p* < 0.001).

**Table 2:** Frequencies of patients whose tears contain, or do not contain IgE and eotaxin. Number of patients (% of patients). Fisher’s exact test, two-tailed: *p* < 0.001

IgE value	Eotaxin value		
	0	Positive	Total
0	11	0	11
	100 %	0 %	100 %
Positive	4	15	19
	21 %	79 %	100 %
Total	15	15	30
	50 %	50 %	100 %

In Fig. 1, mean eotaxin concentration reached 29 ± 8 pg/ml (under the threshold of test sensitivity) (range 0–60 pg/ml) in tears without IgE and 641 ± 154 pg/ml (0–2,132 pg/ml) in tears where IgE was positive (*p* = 0.001).

Tear IgE value was 5.3 ± 3.5 arbitrary U in the 15 patients whose eotaxin concentration was under the threshold of sensitivity (Fig. 2), and 21 ± 4.3 arbitrary U in the 15 patients whose tears contained a positive amount of eotaxin. The difference between the two groups of patients is significant (*p* < 0.001).

Eotaxin concentrations were compared in tears of patients with a history of SAC and patients who had no history of SAC, but were newly diagnosed as suffering from SAC, according to symptoms and signs and after excluding other allergies (Fig. 3). Eotaxin concentration reached 127 ± 47 pg/ml (0–758 pg/ml) in patients with newly diagnosed SAC, and 852 ± 218 pg/ml (0–2,132 pg/ml) in tears of patients where SAC was already known. The difference between both groups is statistically significant (*p* = 0.008).

Concentrations of IgE were not significantly different in patients with a newly diagnosed SAC and those with SAC history (not shown) (*p* = 0.947). Similarly, the clinical score was not significantly different in both groups of patients (Fig. 4) (*p* = 0.797).

**Fig. 5:**
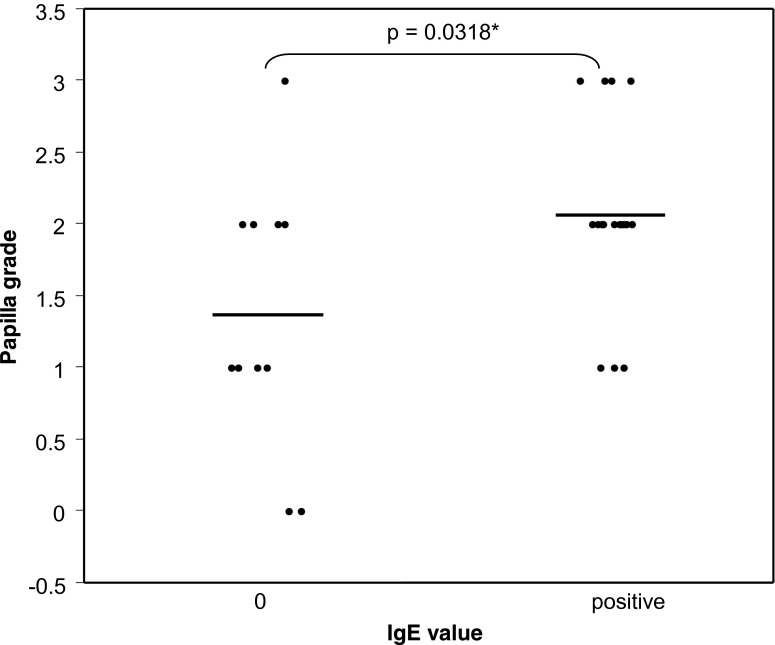
Conjunctival papilla grade and absence (*0*) or presence of IgE in tears. *Bars* = means

Patients which had no IgE in their tears showed a lower conjunctival papilla grade than patients whose tears contained some IgE (Fig. 5) (*p* = 0.0318). On the other hand, there was no correlation between papilla grade and eotaxin presence in tears (not shown) (*p* = 0.2194).

## Discussion

We focused on patients suffering from SAC during the pollen season. SAC mechanism was demonstrated to be different from the mechanisms of more severe allergy cases such as VKC and AKC [3]. For instance, SAC induces penetration of conjunctiva by mast cells and later on neutrophils and eosinophils, whereas VKC and AKC mainly involve T cells and eosinophils [3].

We wanted to specifically study the relation between IgE and eotaxin in tears of patients suffering from SAC during the in season. The relation between IgE and eotaxin has been investigated in plasma of asthmatic patients [17]. Plasma eotaxin values correlated with the total IgE values in asthmatic and non-asthmatic patients, suggesting that eotaxin may play a role in asthma severity [17]. To our knowledge, such comparison was not studied yet in ocular pathologies.

Detection and quantification of IgE in serum is the standard method for diagnosis of seasonal allergy [10]. Some authors have demonstrated that total tear IgE was correlated to serum IgE [7, 10, 18]. On the other hand, the largest contributor to the severity of SAC is the locally produced IgE [19, 20]. Measurement of IgE in tears may be therefore a quicker way to diagnose SAC than measurement of the serum-specific IgE.

A few tests exist to measure total IgE in tears. A test in Japan made it possible to detect high levels of total IgE mean in tears of SAC and PAC patients in comparison to normal patients [7]. A Phadezym–PRIST test was able to show that tears of SAC patients contain more IgE than control tears [19]. The Allerwatch test was used by Japanese groups; tears of an autumnal allergic group of patients contained a higher value of IgE than tears of a control group [21].

For detection of specific IgE in tears, a Japanese group has been evaluating a commercial immunochromatographic test for the semi-quantitative measurement of three specific IgE in tears [22]. For instance, Cedar pollen-specific IgE were significantly higher in tears of allergic conjunctivitis patients than in control subjects.

We chose the Lacrytest since it is an easy and rapid method to estimate total IgE levels in a very small volume of tears. The Lacrytest was previously used in a clinical study on subjects presenting, or not, signs of ocular allergy [23]. The test showed very good results, with a sensitivity of 93.8 % and a specificity of 89.7 %. In another study, if Lacrytest specificity reached 100 % [24], sensitivity was only 20 %. This was probably due to the fact that some patients enrolled in that study did not show ocular symptoms and signs of allergic conjunctivitis at the moment of the visit. Indeed, when patients were submitted to conjunctival provocation test with the suspected allergen, sensitivity raised to 66.7 % [24].

The presence of IgE in tears of patients affected by SAC has already been demonstrated [7, 19]. However, there were always a few patients presenting SAC but where no IgE could be revealed in their tears [19, 25]. For instance, presence of IgE in tears was revealed in 23 out of 28 SAC patients [6]. Why, in our cohort of 30 patients, did only 19 demonstrate significant amounts of total tear IgE? It may be that, by chance, we recruited patients with lower SAC symptoms than in other studies. Therefore, their IgE levels were too low to be revealed by our test. On the other hand, we observed a fair interindividual variance in tear IgE values, phenomenon which was already described [18, 19].

In the present study, we detected 127 ± 47 pg/ml of eotaxin in tears of patients whose pathology was newly described as SAC, and 852 ± 218 pg/ml in tears of patients with a history of SAC. These values are comparable to those reported in tears of SAC patients by Leonardi [15].

Eotaxin has been detected in tears of patients presenting various allergic reactions [15, 26, 27]. This chemokine was also revealed in healthy patients, but to a lesser extent [11]. Eotaxin values in our study are higher than in some other studies. This could be explained by the use of different conditions during the detecting method. An important study compared the amounts of some cytokines and chemokines detected in human tears using the cytometric bead-based assay in different conditions [12]: with or without sample dilution before storage, with or without detergent, with or without bovine serum albumin. In the same initial sample, the authors measured eotaxin values ranging from 170 to 750 pg/ml after applying eight different conditions [12].

We showed, to some extent, a concomitant presence of total tear IgE and tear eotaxin in SAC patients. The absence of IgE in tears necessarily implies the absence of significant amounts of eotaxin. But the presence of tear IgE implies the presence of eotaxin in only 79 % of patients.

In our study, there was no difference in total tear IgE levels between patients with a known SAC and patients with a newly diagnosed SAC. Moreover, the two groups of patients did not differ with regard to their clinical score mean. In contrast, patients with a known SAC exhibited significantly higher amounts of tear eotaxin than patients with a newly diagnosed SAC. Our findings confirm that IgE and eotaxin, if principal actors in the pathology of SAC, act separately along the allergic reaction [1].

Both IgE and eotaxin intervene at different phases of the pathogenesis of SAC. During the phase of sensitization, B lymphocytes of the conjunctiva produce IgE against the allergen, which will link to mast cells. During the challenge phase, the allergen binds with specific IgE on the conjunctival mast cells, leading to mast cell degranulation and mediator release, characteristic of the acute phase allergic reaction. Eotaxin is a major contributor which induces inflammatory cell invasion, driving the late and chronic phase of allergy [2, 3]. This chemokine specifically stimulates eosinophils chemotaxis and aggregation through the chemokine receptor 3 (CCR3), which is expressed in high numbers on eosinophils [28, 29]. Eotaxin induces chemotaxis of other cells wearing CCR3, for instance lymphocytes and basophils [27]. But this CC chemokine also plays a role in mast cell priming, since it provides a co-stimulatory signal for conjunctival mast cells [30].

Extensive studies have proved that experimental allergic conjunctivitis is ablated in mice deficient in eotaxin [1, 3]. After induction of allergic inflammation in the conjunctiva of these mutant mice, the authors counted normal numbers of tissue mast cells in conjunctiva, and measured normal levels of IgE. However, these eotaxin-deficient mice showed a significant impairment of mast cell degranulation and a suppression of clinical symptoms in the acute phase reaction [1]. As mast cell degranulation does not depend on eotaxin, the authors hypothesized that this chemokine might provide a co-stimulatory signal, and that CCR3 plays a major role in activation of mature connective tissue-type mast cells in ocular tissue. Indeed, when CCR3 was blocked, allergen-mediated hypersensitivity reaction and IgE-mediated mast cell degranulation were suppressed [1]. Therefore, the eotaxin/CCR3 axis is the main control of mast-cell-mediated allergy.

The role of CCR3 in allergic conjunctivitis has been previously demonstrated in mice subjected to allergic sensitization [31]. Anti-CCR3 suppressed both clinical signs of allergic inflammation and mast cell degranulation, but could not reduce the serum levels of specific IgE in mice [31].

Presence of conjunctival papillae is characteristic of AKC, VKC, and contact lens wear [13, 16, 32, 33]. We were able to demonstrate that our SAC patients had a low to moderate conjunctival papilla grade during the in season. We observed that patients with a positive amount of IgE in their tears were characterized by a higher conjunctival papilla grade than patients with no IgE. A mild papillary hypertrophy was previously observed in the upper tarsal conjunctiva of patients suffering from seasonal allergic rhinoconjunctivitis [4].

In conclusion, we investigated a cohort of patients suffering from SAC during the pollen season. We are aware of some limitations of interpretation of our data; Lacrytest is a semi-quantitative test with an arbitrary endpoint to determine positive or negative results, and the detecting test for eotaxin has a sensitivity threshold of 60 pg/ml. However, the data suggest that when patient tears contained IgE, they were often likely to contain eotaxin too. Patients with a history of SAC were compared to patients with a newly diagnosed SAC. Both groups of patients demonstrated the same level of total tear IgE in mean, and the same clinical score. In contrast, patients with a known SAC had a significantly higher level of eotaxin in their tears than patients with a newly SAC. This confirms that, if IgE and eotaxin are principal actors in IgE-mediated SAC, they contribute separately to the reaction.
